# Persistence of human respiratory viral RNA in wastewater-settled solids

**DOI:** 10.1128/aem.02272-23

**Published:** 2024-03-19

**Authors:** Mengyang Zhang, Laura Roldan-Hernandez, Alexandria Boehm

**Affiliations:** 1 Department of Civil and Environmental Engineering, School of Engineering and Doerr School of Sustainability, Stanford University, Stanford, California, USA; Centers for Disease Control and Prevention, Atlanta, Georgia, USA

**Keywords:** human respiratory viruses, persistence, wastewater solids, wastewater-based epidemiology

## Abstract

Wastewater-based epidemiology has emerged as a valuable tool for monitoring respiratory viral diseases within communities by analyzing concentrations of viral nucleic-acids in wastewater. However, little is known about the fate of respiratory virus nucleic-acids in wastewater. Two important fate processes that may modulate their concentrations in wastewater as they move from household drains to the point of collection include sorption or partitioning to wastewater solids and degradation. This study investigated the decay kinetics of genomic nucleic-acids of seven human respiratory viruses, including severe acute respiratory syndrome coronavirus 2 (SARS-CoV-2), respiratory syncytial virus (RSV), human coronavirus (HCoV)-OC43, HCoV-229E, HCoV-NL63, human rhinovirus (HRV), and influenza A virus (IAV), as well as pepper mild mottle virus (PMMoV) in wastewater solids. Viruses (except for PMMoV) were spiked into wastewater solids and their concentrations were followed for 50 days at three different temperatures (4°C, 22°C, and 37°C). Viral genomic RNA decayed following first-order kinetics with decay rate constants *k* from 0 to 0.219 per day. Decay rate constants *k* were not different from 0 for all targets in solids incubated at 4°C; *k* values were largest at 37°C and at this temperature, *k* values were similar across nucleic-acid targets. Regardless of temperature, there was limited viral RNA decay, with an estimated 0% to 20% reduction, over the typical residence times of sewage in the piped systems between input and collection point (<1 day). The *k* values reported herein can be used directly in fate and transport models to inform the interpretation of measurements made during wastewater surveillance.

**IMPORTANCE:**

Understanding whether or not the RNA targets quantified for wastewater-based epidemiology (WBE) efforts decay during transport between drains and the point of sample collection is critical for data interpretation. Here we show limited decay of viral RNA targets typically measured for respiratory disease WBE.

## INTRODUCTION

Wastewater-based epidemiology uses concentrations of infectious disease targets, typically genomic nucleic-acids, in wastewater to infer information about disease occurrence in the contributing community. Research has shown that respiratory diseases such as those caused by severe acute respiratory syndrome coronavirus 2 (SARS-CoV-2) (1–8), influenza (9–12), respiratory syncytial virus (RSV) (9, 12, 13), rhinovirus (12), and seasonal coronaviruses (12) can be tracked by measuring concentrations of their genomic nucleic-acids in wastewater. However, little is known about the fate of viral genomic nucleic-acids in wastewater. Two important fate processes that may modulate their concentrations in wastewater as they move from household or building drains to the point of sample collection include sorption or partitioning to wastewater solids (14) and degradation (7).

Research and observational studies in wastewater indicate that viral targets tend to partition to the solid phase of wastewater. Yin et al. (15, 16) compiled data from the literature showing a high degree of enrichment of a variety of non-enveloped human viruses in wastewater solids, and conducted laboratory studies to document adenovirus partitioning to wastewater solids. Ye et al. (17) conducted laboratory studies and found that enveloped viruses tended to partition to wastewater solids more so than non-enveloped viruses. Roldan-Hernandez et al. (18) conducted laboratory and field studies and found that a range of respiratory viruses, including SARS-CoV-2, RSV, rhinovirus, as well as bacteriophage F+ coliphage/MS2, tended to partition to solids with partitioning coefficients of 10^3^ to 10^4^ mL/g. Given the enrichment consistently observed in wastewater solids across a range of targets (10, 19), wastewater solids represent an important matrix for wastewater disease surveillance (20).

Degradation of viral nucleic-acids in wastewater may also be an important fate process affecting their concentrations in wastewater. There are limited studies on the degradation of viral nucleic-acids in wastewater, particularly for respiratory viral nucleic-acids. A number of studies have documented the persistence of SARS-CoV-2 RNA in the liquid phase of wastewater (21). First-order decay rate constants of 0.04–0.29 day^−1^ were reported for SARS-CoV-2 RNA in wastewater influent with the highest decay rate constant observed at the higher tested temperature (37°C) (22, 23). Only one study (24) has measured the persistence of SARS-CoV-2 RNA in wastewater solids; they observed first-order decay rate constants ranging from 0.01 to 0.10 day^−1^ (24). Beyond SARS-CoV-2, there is a notable lack of decay kinetics data for human respiratory viral nucleic-acids in wastewater liquids or solids.

In this study, we fill this knowledge gap by documenting the persistence of human respiratory viral RNA in wastewater solids by measuring the RNA decay kinetics for seven human respiratory viruses, including SARS-CoV-2, RSV, seasonal human coronavirus OC43 (HCoV-OC43), HCoV-229E, HCoV-NL63, human rhinovirus (HRV), and influenza A virus (IAV), as well as pepper mild mottle virus (PMMoV). PMMoV was monitored because it is often used as fecal strength control and endogenous viral process control (25). Viruses (except for PMMoV) were spiked into the wastewater solids. The effect of temperatures (4°C, 22°C, and 37°C) on decay was investigated. The decay rate constants provided herein for viral RNA in wastewater solids can be used directly in fate and transport models to inform the interpretation of measurements made during wastewater surveillance (7).

### Experimental methods

#### Overview

The decay of genomic RNA from seven human respiratory viruses, including SARS-CoV-2, RSV, HCoV-OC43, HCoV-229E, HCoV-NL63, HRV, and IAV, in wastewater solids was monitored using reverse-transcriptase-droplet digital polymerase chain reaction (RT-ddPCR) over 50 days. Wastewater solids were spiked with a purified cocktail of heat- or chemically-inactivated viruses. The concentration and decay of endogenous PMMoV were monitored concurrently. Intrinsic biological characteristics of the viruses are shown in Table 1. Duplicate experiments were conducted at three temperatures 4°C, 22°C, and 37°C, representing temperatures that wastewater may experience in cold, temperate, and tropical regions, respectively.

**TABLE 1:** Characteristics of targeted human respiratory viruses^*a*^

Virus	Family/genus	Structure	Gene type	Genome size (kb)	Virion size (nm)	Shape
SARS-CoV-2 (26)	Coronaviridae/ Betacoronavirus	Enveloped	+ssRNA	30	50–140	Spherical
RSV (27)	Pneumoviridae/ Orthopneumovirus	Enveloped	-ssRNA	15	150–250	Spherical, filamentous, asymmetric
HCoV-OC43 (28)	Coronaviridae/ Betacoronavirus	Enveloped	+ssRNA	31.5	80–120	Spherical or pleomorphic
HCoV-229E (28)	Coronaviridae/ Alphacoronavirus	Enveloped	+ssRNA	27.5	80–120	Spherical or pleomorphic
HCoV-NL63 (28)	Coronaviridae/ Alphacoronavirus	Enveloped	+ssRNA	27.5	80–120	Spherical or pleomorphic
HRV (29)	Picornavirus/ Enterovirus	Non-enveloped	+ssRNA	7	23–28	Icosahedral
IAV (30)	Orthomyxoviridae/ Alphainfluenzavirus	Enveloped	Segmented -ssRNA	13.5	80–120	Spherical
PMMoV (31)	Virgaviridae/ Tobamovirus	Enveloped	+ssRNA	6.35	~312 in length	Rod-shaped

^*a*^ “kb” is kilobases.

#### Wastewater solids sample collection

A 24-hour composite primary sludge sample (500 mL gathered every 4 hours over 24 hours) was collected from the San José–Santa Clara Regional Wastewater Facility and transported to the lab on ice within 2 hours, which is subsequently referred to as “wastewater solids” sample. The wastewater solids sample was aliquoted into sterile 50 mL centrifuge tubes and spiked with the human respiratory virus cocktail (for details see Persistence Experiment Setup).

#### Propagation of HCoV-OC43

Vero E6 cells (CRL-1586, ATCC) were purchased from ATCC (Manassas, Virginia), and maintained in Dulbecco’s modified Eagle’s medium (DMEM) supplemented with 10% fetal bovine serum (FBS) and 1% Penicillin-Streptomycin (P/S) and incubated at 37℃/5% CO_2_ until confluence. HCoV-OC43 was provided by Dr. Jeffrey S. Glenn (Stanford University, Stanford, CA). For virus propagation, HCoV-OC43 was inoculated onto confluent Vero E6 cells, which was maintained in serum-free DMEM, at a multiplicity of infection of 0.01. Infected cells were incubated at 37℃/5% CO_2_ until a significant cytopathic effect was observed, which usually took 6 days. Virus suspension was collected and centrifuged at 4,000 *× g* for 10 minutes. Then, the supernatant was filtered through 0.22 µm-pore size membranes to remove cell debris. The HCoV-OC43 stock was stored at −80°C until further purification (see Virus Purification section) and used in persistence experiments.

#### Virus purification

Heat-inactivated SARS-CoV-2 (Isolate: USA-WA1/2020, 0810587CFHI, ZeptoMetrix), heat-inactivated HCoV-229E (0810229CFHI, ZeptoMetrix), heat-inactivated HCoV-NL63 (0810228CFHI, ZeptoMetrix), chemical-inactivated IAV (Isolate: A/Singapore/63/04, NATFLUAH1(2009)-STQ, ZeptoMetrix), infectious RSV-A (Isolate: 2006 Isolate, 0810040ACF, ZeptoMetrix), and infectious HRV-B14 (0810284CF, ZeptoMetrix) were purchased from ZeptoMetrix (Buffalo, New York). HCoV-OC43 was propagated in the lab as described above. SARS-CoV-2, HCoV-229E, and HCoV-NL63 were suspended in viral culture fluids and inactivated at 55°C–60°C for 1 hour by the manufacturer prior to shipment. The manufacturer does not release details on the methods of chemical inactivation for IAV, but it is provided as purified and suspended in a proprietary matrix. Infectious RSV-A, HRV, and HCoV-OC43 were heat-inactivated in our laboratory to address biosafety concerns. HCoV-OC43 was suspended in a cell culture medium and inactivated at 55°C for 1 hour. RSV and HRV were suspended in viral culture fluids and inactivated at 56°C for 5 minutes (32) and 16 minutes (33), respectively, following the Pathogen Safety Data Sheets guidelines, as required by Stanford Biosafety. Inactivated SARS-CoV-2, RSV, HCoV-OC43, HCoV-229E, HCoV-NL63, and HRV stocks were subsequently purified using Amicon Ultra-0.5 mL centrifugal filters (100 kDa MWCO; Millipore UFC5100). Briefly, 500 µL of each virus stock was centrifuged at 14,000 × *g* for 20 minutes. The retentate was then immediately recovered by centrifugation at 1,000 × *g* for 2 minutes, with the filter inverted. Each purified virus stock was diluted between 1:1 and 1:100 with autoclaved phosphate-buffered saline (PBS, VWR, US) to achieve a final concentration of approximately 10^6^–10^7^ gene copies/mL. A volume of 480 µL of each diluted purified virus stock, including SARS-CoV-2, RSV, HCoV-OC43, HCoV-229E, HCoV-NL63, HRV, and IAV, were combined into a single virus cocktail, 3,360 µL total volume, used to spike into wastewater solids samples.

#### Persistence experiment setup

The wastewater solids sample was mixed thoroughly by inverting 4–5 times and 5 mL aliquots were placed into 46 sterile 50 mL centrifuge tubes. Hereafter, each separate tube is referred to as a subsample. Two subsamples were set aside without adding a virus cocktail to measure background concentrations of targeted viral RNA. Seventy microliters of the virus cocktail were spiked into each of the remaining 44 subsamples and mixed by vortexing for 10 seconds. Forty-two subsamples spiked with viruses were then placed in constant-temperature rooms at 4°C, 22°C, and 37°C while being covered by opaque boxes to shield them from light. Two subsamples were sacrificed at each time point (5, 10, 15, 20, 30, 40, and 50 days) for each temperature (4°C, 22°C, and 37°C). The remaining two subsamples spiked with viruses were sacrificed immediately and served as the day 0 sample for all temperature conditions. To ensure proper equilibration of the day 0 subsamples, before proceeding to pre-analytical processing and RNA extraction, day 0 subsamples were mixed at ~20 rpm using a tube roller (Globe Scientific, GSCIGTR-AVS) at 4°C for 3 hours (18). The equilibrated day 0 subsamples and subsamples used for measuring background viral RNA concentrations were subsequently subjected to pre-analytical processing and RNA extraction within 1hour.

#### Pre-analytical processing

Each subsample was processed as follows. The subsample was centrifuged at 24,000 × *g* for 15 minutes at 4°C and the supernatant was discarded. Approximately 0.25 g wet weight of the dewatered solids (the pellet) was suspended in a DNA/RNA shield (R1100-250, Zymo Research) to achieve a final concentration of 75 mg wet weight mL^−1^; this concentration of solids used at this step in the process has been shown to alleviate inhibition in the downstream RT-ddPCR (34). The mixture was homogenized with five grinding balls (OPS DIAGNOSTICS, GBSS 156-5000-01) at 6.5 m s^−1^ for 1 minute using the MP Bio Fastprep-24TM (MP Biomedicals, Santa Ana, CA), followed by a centrifugation at 3,500 × *g* for 30 seconds. Two hundred microliters of the supernatant were then used for RNA Extraction immediately and the rest was stored at −80°C as back-up.

Positive and negative control was included in each batch of samples subjected to pre-analytical processing and RNA extraction (usually six subsamples in a batch). The positive control consisted of dewatered wastewater solids (not spiked with human viruses) suspended in a DNA/RNA shield containing bovine coronavirus (BCoV, CALF-GUARD, Zoetis, Parsippany-Troy Hills, NJ; 1.5 µL of BCoV per mL of DNA/RNA shield) at a concentration of 75 mg wet weight/mL. The negative control consisted of a DNA/RNA shield. The recovery efficiency for each batch of RNA extraction was determined by calculating the ratio of the recovered spiked-in internal control of BCoV to the initially added amount of BCoV.

The percent solids were determined for each subsample as follows. A portion of dewatered wastewater solids (not spiked with virus cocktail) was heated at 105°C for 24 hours. The percentage of solids was determined by comparing the solid’s weight before and after heating (Table S1). This value is needed in order to express measurements in units of copies of the target per dry weight of solids.

#### RNA extraction

Two hundred microliters of supernatant were added to the Allprep PowerViral DNA/RNA extraction kit (28000-50, Qiagen) and further purified with the OneStep PCR inhibitor removal columns (D6030, Zymo Research), following the manufacturer’s instructions. Six subsamples at each time point (two subsamples at each temperature of 4°C, 22°C, and 37°C) were sacrificed and proceeded to RNA extraction as the same extraction batch. Each RNA extract (~100 µL) was aliquoted into five 100 µL-PCR LoBind tubes and stored at −80°C until quantification (typically between 10 and 60 days).

#### RT-ddPCR

SARS-CoV-2 and RSV, IAV, and HRV genomic RNA were quantified using duplex assays, respectively. HCoV-OC43, HCoV-229E, and HCoV-NL63 were quantified using a triplex assay. PMMoV and BCoV were quantified using singleplex assays separately. Primer and probe sequences, as well as the target gene and the size of the amplicon, are provided in Table S2, and all represent previously published assays. All RT-ddPCR assays were performed using the OneStep RT-ddPCR Advanced kit for probes (1864021, BioRad). Each 22 µL of reaction mix contained 5.5 µL of RNA template, 5.5 µL of Supermix, 2.2 µL of 200 U µL^−1^ Reverse Transcriptase (RT), 1.1 µL of 300 mM dithiothreitol (DTT), 2.2 µL of nuclease-free water, and 5.5 µL of primer and probe mixture. For singleplex assays and duplex assays, the final concentrations of primer and probe were 0.9 µM and 0.25 µM, respectively. In the case of multiplexing HCoV-229E (fluorescent dye fluorescein amidite, FAM), HCoV-NL63 (fluorescent dye hexachloro-fluorescein, HEX), and HCoV-OC43 (FAM/HEX) using the triplex probe mixing approach, the primer concentrations for HCoV-OC43, HCoV-229E, and HCoV-NL63 were 0.9 µM, and the probe concentrations for HCoV-229E and HCoV-NL63 were 0.25 µM, while the probe concentration for HCoV-OC43 was 0.125 µM for each dye (FAM and HEX). The template was run neatly for all assays. Droplets were generated using the AutoDG Automated Droplet Generator (Bio-Rad, Hercules, CA), and RT-PCRs performed utilizing the C1000 Touch Thermal Cycler (Bio-Rad). The thermal cycling conditions for each RT-ddPCR assay are shown in Table S3. After amplification, the droplets were analyzed using the QX200 droplet reader (Bio-Rad) and the QX Manager software. Three triplicate wells were run for each RNA extract. Synthetic viral RNA or RNA extracted from virus stock (Table S2) and nuclease-free water were used as positive and negative PCR controls, respectively, with three replicates on each PCR plate.

Inhibition was assessed using a serial dilution of RNA extract from a virus-spiked wastewater solids sample for SARS-CoV-2, RSV, HCoV-OC43, HCoV-229E, HCoV-NL63, HRV, and PMMoV. For IAV, the synthetic RNA was added to RNA extract from a wastewater solids sample (with no virus spiked) and then diluted as a series due to the low concentration of IAV stock. Comparison between the viral RNA concentration measured obtained from the undiluted template and those obtained using diluted template (series dilution of 1:1, 1:2, 1:5, 1:10 for SARS-CoV-2 and RSV; series dilution of 1:1, 1:5, 1:25, 1:125 for HCoV-OC43, HCoV-229E, HCoV-NL63, HRV, IAV, and PMMoV) served as the basis for evaluating inhibition.

#### Dimensional analysis

The threshold for RT-ddPCR was manually set using the QX Manager software, six wells were merged for analysis for each time point for each temperature (two replicate subsamples, each run in triplicate). The concentration of the targeted viral RNA was converted to concentrations per dry weight of solids in units of copies per gram of dry weight (cp g^−1^) following equation (14):


(1) $$C=\left(\frac{c\;\;V_{rxn}}{V_{template}}V_{extract}\right)/\left(M_{wet}P\right)$$

where *C* represents the viral RNA concentration in dry solids with the unit of cp g^−1^, *c* represents the concentration from QX Manager software with the unit of copies µL^−1^, *V_rxn_* represents the volume of RT-ddPCR reaction mix with 20 µL per well, *V_template_* represents the volume of RNA template in RT-ddPCR reaction mix with 5 µL per reaction, *V_extract_* represents the volume of RNA extracts with 100 µL per extract, *M_wet_* represents the mass of wet solids used for each RNA extraction with 0.015 g per RNA extraction (200 µL of DNA/RNA shield suspension with solids concentration of 75 mg mL^−1^), *P* represents the percent of solids (Table S1).

The theoretical lower detection limit, 5.12 × 10^3^ cp g^−1^, was calculated as previously described by Kim et al. (35), which is three positive droplets total across six merged wells out of 20,000 accepted droplets in each well. Data from wells with less than 10,000 total accepted droplets were not included in the analysis. The error was reported as the 68% confidence interval by the QX Manager software.

#### Decay rate constants

The first-order decay rate constants (*k*) of viral RNA in wastewater solids were estimated by fitting a first-order decay model to the corresponding persistence data:


(2) $$ln\left(\frac{C}{C_{0}}\right)=\beta_{0}-kt+\varepsilon$$

where *C* is the concentration of viral RNA in dry solids at each time point (cp g^−1^), which was calculated as described in the Dimensional Analysis Section; *C_0_* is the average concentration of viral RNA in dry solids at 0 day (cp g^−1^); *β*_0_ is the intercept, *k* is the first-order decay rate constant (day^−1^), *t* is the incubation time (day), and ε is the residual standard error of the model. Data under the theoretical lower detection limit was excluded from the linear regression. Data from each temperature condition was considered separately in equation 2. *k* and its standard error were determined using the function “lm” in R (version 4.2.2) and Rstudio (Version 2022.12.0+353), and the goodness of fit of the model was assessed through examination of the coefficient of determination (R^2^). We used *P* < 0.05 to indicate a coefficient significantly different from 0.

The standard deviation of $ln(\frac{C}{C_{0}})$ was estimated by propagating error on the numerator and the denominator (36). To do so, we assumed that the error on the numerator and denominator were normal and equal to the larger of the upper or lower error on the measurement, as it is not symmetric. The standard deviation was used in plotting the data.

The time required for a 90% reduction in viral RNA in wastewater solids (T_90_) was calculated as follows:


(3) $$T_{90}=-\frac{ln\left(0.1\right)}{k}$$

where *k* is the first-order decay rate constant (day^−1^).

#### Statistical analysis

We tested whether the decay rate constants of viral RNA in wastewater solids were significantly affected by temperature or viral species using a multiple linear regression model (37). We used the “lm” function in R following the model (equation 4):


(4) $$ln\left(\frac{C}{C_{0}}\right)=\beta_{0}+\beta_{1}t+\sum_{i}^{n}\beta_{i}X_{i}+\varepsilon$$

where *β*_0_ is the intercept; *β*_1_ and *β*_*i*_ are the regression coefficients for time *t* (day) and each factor *X_i_*, respectively; ε is the residual standard error of the model. The factors *X_i_* include virus species dummy variables (categorical) and temperature (continuous). Post hoc Tukey contrasts were used to test whether the decay of viral RNA differed significantly among virus species using the “glht” function in R. *P* < 0.05 indicates a significant impact factor for viral RNA decay.

Tukey’s honestly significant difference (HSD) test was used to test whether the decay of two viruses at the same temperature (4°C, 22°C, or 37°C) was different from each other (38). *P* < 0.05 indicates a significant difference between the decay of two viruses at the same temperature.

All data from this study (time series of measured concentrations and calculated *k* values) are available in the Stanford Digital Repository DOI: https://purl.stanford.edu/px913dv7681.

## RESULTS

### QA/QC

The results of this study are reported following the Environmental Microbiology Minimum Information guidelines (39). The positive and negative extraction and RT-ddPCR controls were all positive and negative, respectively. BCoV recoveries for all batches of RNA extraction were ~56%–115% with a median recovery of 88% (Table S4) suggesting good recovery of RNA. No attempt was made to adjust virus concentrations based on recovery due to the complexities associated with estimating viral RNA recovery using surrogate viruses (40). We tested serial dilutions of extracted viral RNA from viral cocktail spiked wastewater solids sample as a template in RT-ddPCR to test for inhibition. The results indicate no inhibition (Fig. S1) as concentrations measured using a diluted template, adjusted for the dilution, were similar to those obtained using an undiluted template.

### Decay of human respiratory viral RNA in wastewater solids

Purified human viral cocktail, including SARS-CoV-2, RSV, HCoV-OC43, HCoV-229E, HCoV-NL63, HRV, and IAV, was spiked into wastewater solids and their concentrations were monitored over 50 days at 4°C, 22°C, or 37°C. The background viral RNA concentrations in the solids sample were within two orders of magnitude of our detection limit (Table 2) for all human respiratory viruses tested, consistent with expectations based on pilot measurements. Given our desire to be able to observe logarithmic decay of the viral RNA in these experiments, spiking of exogenous virus into the experiments was required. The concentrations in the spiked subsamples at *t* = 0 of SARS-CoV-2, RSV, HCoV-OC43, HCoV-229E, HCoV-NL63, and HRV were ~0.51–2.0 × 10^7^ copies per gram dry weight of wastewater solids, while the initial average concentration of IAV was 9.3 × 10^4^ cp g^−1^ and was limited by the low concentration of IAV stock (Table 2). Therefore, spiking the samples with viruses raised the concentrations by orders of magnitude (Table 2).

**TABLE 2:** Background viral RNA concentrations in wastewater solids and *t* = 0 day sample^*a*^

Virus	Background concentration (cp g^−1^)	Day 0 concentration (cp g^−1^)	Percentage (%)
RSV	5.51 × 10^3^	5.13 × 10^6^	0.11
SARS	1.25 × 10^5^	6.58 × 10^6^	1.90
OC43	0.00	1.11 × 10^7^	0.00
NL63	9.67 × 10^3^	2.09 × 10^7^	0.05
229E	1.35 × 10^4^	1.37 × 10^7^	0.10
IAV	1.26 × 10^4^	9.28 × 10^4^	13.53
HRV	2.80 × 10^5^	6.19 × 10^6^	4.52
PMMoV	4.36 × 10^8^	4.36 × 10^8^	–

^*a*^ cp g^−1^ represents viral RNA copies per gram dry weight of solids. The percentage is reported as a concentration in the background divided by the concentration of *t* = 0 sample.

**Fig 1:**
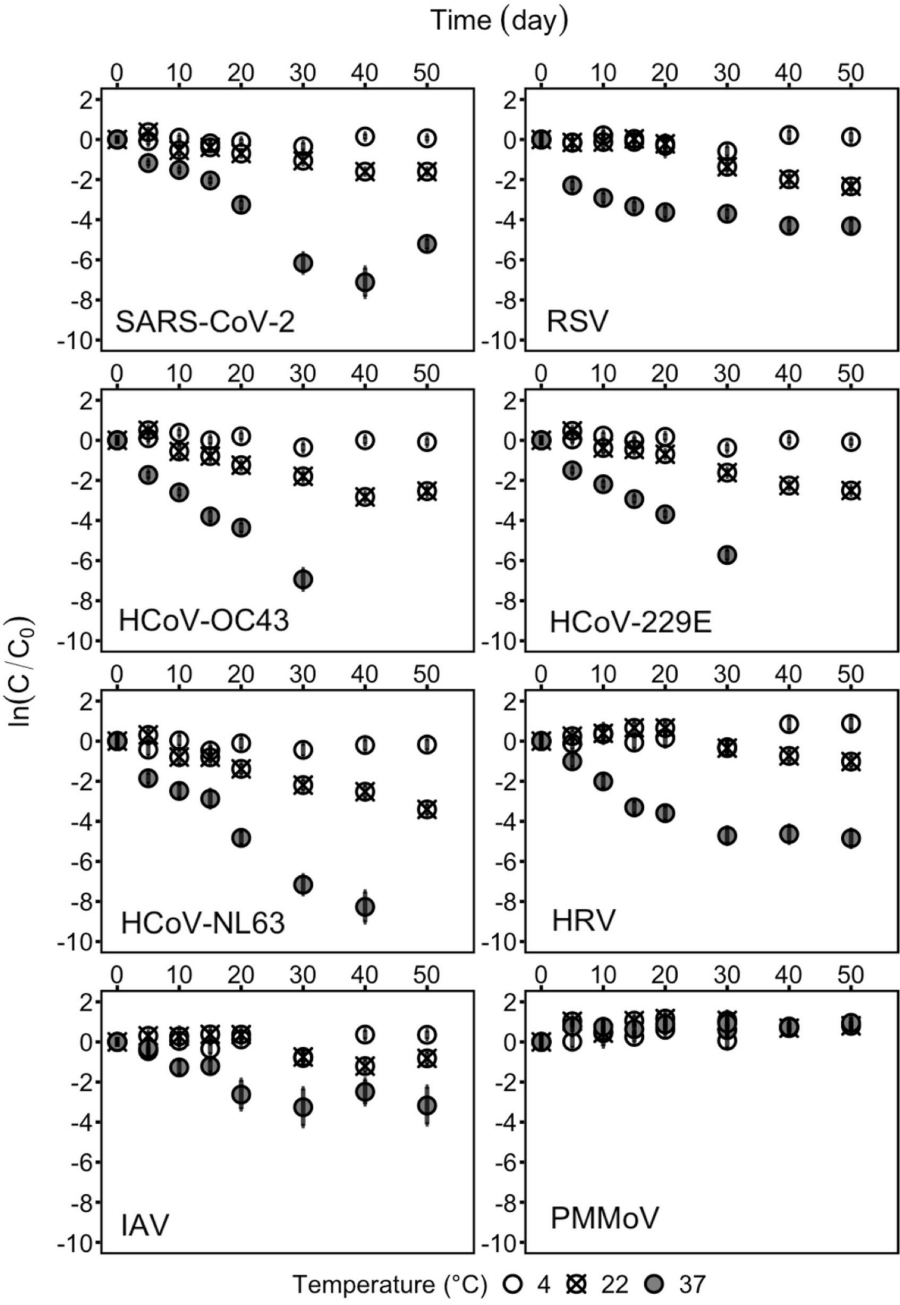
Decay of spiked-in human respiratory viral RNA and endogenous PMMoV RNA in wastewater-settled solids. Each data point was merged from six RT-ddPCR wells. The error bar represents the measurement standard deviation.

The decay of human respiratory viral RNA is shown in Fig. 1, and first-order decay rate constants *k* and T_90_ values are summarized in Table 3. Very little decay was observed for all targeted human respiratory viral RNA in wastewater solids at 4°C even after 50 days and their first-order decay rate constants were not significantly different from 0 (*P* > 0.05). Human viral RNA decayed in the wastewater solids at 22°C with *k* values ranging from 0.028 to 0.072 day^−1^; HRV had the lowest *k* of 0.028 ± 0.009 day^−1^ and HCoV-NL63 with the highest *k* of 0.072 ± 0.006 day^−1^ at 22°C (errors represent standard error). Overall, human respiratory viral RNA decayed faster at 37°C than at 22°C, ranging from 0.064 to 0.219 day^−1^. RSV and IAV RNA decayed the slowest at 37°C with *k* values of 0.066 ± 0.019 day^−1^ (RSV) and 0.064 ± 0.014 day^−1^ (IAV). R^2^ values for models where there was significant decay (*k* different from 0) varied from 0.60 to 0.99 (*P* < 0.05) suggesting first-order decay (equation 2) is a reasonable model for the observed decay.

**TABLE 3:** Human respiratory viral RNA and PMMoV RNA first-order decay rate constants (*k*) with standard error (SE) and T_90_ in wastewater-settled solids^*a*^

Virus	Temperature (°C)	*k* (day^−1^) (SE)	R^2^	T_90_ (day)
SARS-CoV-2	4	−0.001 (0.004)^*a*^	0.023	
	22	0.038 (0.005)	0.90	60.6
	37	0.133 (0.026)	0.81	17.3
RSV	4	−0.002 (0.006)^*a*^	0.015	
	22	0.053 (0.007)	0.89	43.4
	37	0.066 (0.019)	0.68	34.9
HCoV-OC43	4	0.005 (0.004)^*a*^	0.20	
	22	0.064 (0.008)	0.91	36.0
	37	0.219 (0.012)	0.99	10.5
HCoV-229E	4	0.004 (0.004)^*a*^	0.16	
	22	0.060 (0.006)	0.95	38.4
	37	0.180 (0.010)	0.99	12.8
HCoV-NL63	4	0.001 (0.005)^*a*^	0.01	
	22	0.072 (0.006)	0.96	32.0
	37	0.208 (0.015)	0.98	11.1
HRV	4	−0.017 (0.008)^*a*^	0.46	
	22	0.028 (0.009)	0.60	82.2
	37	0.095 (0.018)	0.83	24.2
IAV	4	−0.009 (0.009)^*a*^	0.14	
	22	0.030 (0.009)	0.66	76.8
	37	0.064 (0.014)	0.77	36.0
PMMoV	4	−0.013 (0.008)^*a*^	0.35	
	22	−0.007 (0.009)^*a*^	0.10	
	37	−0.010 (0.005)^*a*^	0.34	

^*a*^ Indicates that the slope is not significant from zero; for these values, a T_90_ is also not provided.

T_90_ values for the viruses were largest at 4°C and smallest at 37°C (Table 3). Notably, at 4°C, T_90_ could not be calculated due to non-significant *k* values for all human respiratory viral RNA targets. At 22°C, T_90_s ranged from 32.0 to 38.4 days for the HCoVs and extended to 43.4–82.2 days for other viruses. It is noteworthy that over the 50-day experimental period, less than 1 log_10_ reduction was observed for SARS-CoV-2, IAV, and HRV at 22°C. At 37°C, T_90_s ranged from 10.5 to 36.0 days with the smallest T_90_s observed for HCoVs and the largest for RSV and IAV.

### Decay of endogenous PMMoV RNA in wastewater solids

The background or initial RNA concentration of endogenous PMMoV was 4.36 × 10^4^ cp g^−1^, which is consistent with that measured previously in the same wastewater treatment plant (24). No significant decay was observed for PMMoV in wastewater solids at all temperatures of 4°C, 22°C, and 37°C, as all *k* values were not significant from 0 (Table 3).

### Impact of virus species and temperature on viral RNA decay in wastewater solids

The impact of virus species and temperature on the decay of viral RNA in wastewater solids was evaluated by a multiple linear regression following equation 4. The coefficients of the continuous variables time and temperature coefficients were significantly different from 0 in the model (*P* < 0.001, coefficients summarized in Table S5). For the dummy virus species variables, only the PMMoV dummy variable coefficient was significantly different from 0 (*P* < 0.001, Table S5). The coefficient of determination (R^2^) for the multiple linear regression model was 0.54 (*P* < 2.2 × 10^−16^). Post hoc comparisons indicate that the decay of PMMoV RNA was significantly different and slower than the other seven targeted human respiratory viral RNA (*P* < 0.01, Table S6). For the rest of the 21 viral species binary comparisons, only the decay of IAV and HCoV-NL63 was significantly different from each other (*P* = 0.03) with decay slower for IAV.

The decay kinetics of viral RNA exhibited non-significant variations at 4°C in wastewater solids across different virus species as their first-order decay rate constants were all non-significant form 0. Based on the results of Tukey’s HSD test (Table S7), PMMoV RNA showed slower decay than SARS-CoV-2, HCoV-OC43, HCoV-229E, HCoV-NL63, RSV, and IAV at either 22°C or 37°C (*P* < 0.01). PMMoV RNA did not show significantly different decay from HRV RNA at 22°C (*P* = 0.054) but exhibited significantly slower decay than HRV RNA at 37°C (*P* < 0.01). Notably, there was no significant difference between the decay for three HCoVs at all temperature conditions (*P* > 0.05). HCoV-NL63 RNA showed significantly faster decay than IAV at both 22°C and 37°C (*P* < 0.01).

## DISCUSSION

**Fig 2:**
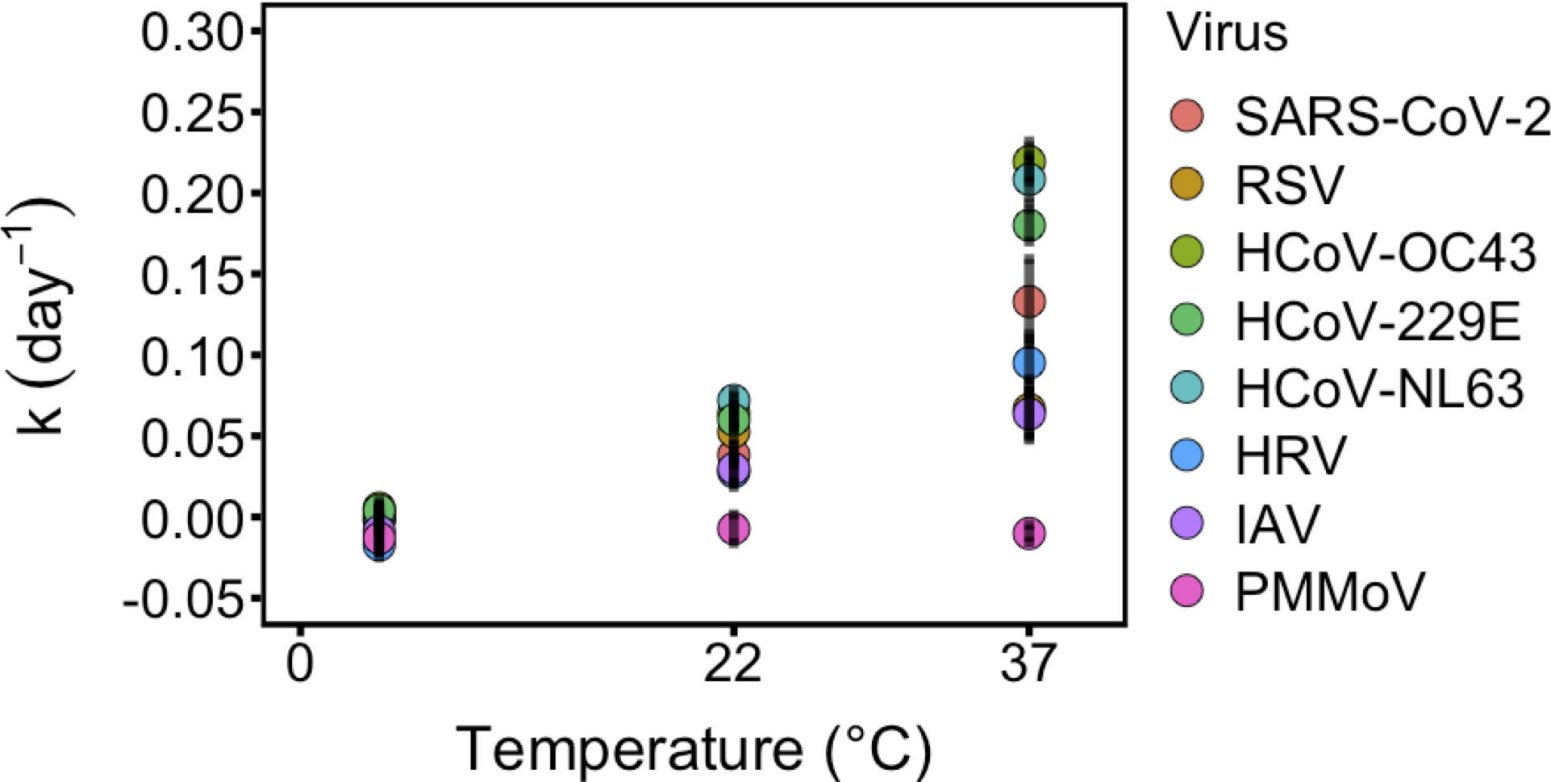
First-order decay rate constants of spiked-in human respiratory viral RNA and endogenous PMMoV RNA in wastewater-settled solids at different temperatures. Error bars represent the standard error.

This study provides decay kinetics of SARS-CoV-2, RSV, HCoV-OC43, HCoV-229E, HCoV-NL63, HRV, and IAV RNA in wastewater solids. We found limited decay of the targets over the 50-day experimental period particularly at temperatures of 4°C and 22°C. Consistent with findings with other pathogens and infectious disease targets (41, 42), we found that decay rates increase with higher temperatures (Fig. 2).

While previous observation studies have shown a correlation between the respiratory virus nucleic-acid concentrations in wastewater solids and disease occurrence in communities contributing to the wastewater stream (12), there was no data on the decay of these infectious disease targets in wastewater, aside from SARS-CoV-2. Therefore, the new data presented here fills an important knowledge gap. The decay rate constants represent an important parameter for fate and transport models that may ultimately inform the interpretation of measurements made during wastewater surveillance. For example, the fate and transport model described by Wolfe et al. (7) related the number of people shedding viral nucleic-acids into wastewater to their concentrations in wastewater solids and requires first-order decay rate constants as input parameters. Based on the *k* values measured herein, limited to no decay of the infectious disease targets is expected over the time scales sewage typically spends in a wastewater sewage system (<24 hours); estimated 0% to 20% reduction over 24 hours depending on target and temperature. This finding suggests that incorporating viral target decay may not be imperative in environmental models that link the shedding from infected individuals to the anticipated concentrations at wastewater solids collection sites, thereby simplifying data interpretation (7, 43). Additional work in actual sewer networks may be needed to further confirm this (44–46).

Another practical concern for wastewater surveillance programs is the effect of storage on infectious disease targets in samples. Based on our results, prolonged storage at 4°C for up to 50 days will have a negligible impact on concentrations of the tested viral targets. Biobanking of samples for retrospective studies or analysis is imperative for understanding the emergence of new viral outbreaks, or new variants or subtypes. Even at room temperature, less than 1 log_10_ decay was observed over periods of up to 1 month.

There are no estimates of *k* in previous literature for the studied respiratory virus targets in wastewater solids to compare our results with, except SARS-CoV-2. Roldan-Hernandez et al. (24) documented *k* values for indigenous SARS-CoV-2 RNA in wastewater solids; they found first-order *k* values of 0.011–0.036 day^−1^, 0.021–0.089 day^−1^, and 0.047–0.091 day^−1^ for experiments conducted at 4°C, 22°C, and 37°C, respectively (24). These values are similar to the ones reported herein obtained with heat-inactivated SARS-CoV-2 spiked into the wastewater solids. The similar decay rate constants suggest similar decay kinetics of indigenous and spiked in SARS-CoV-2 RNA, and this finding might extend to the other viral targets. As the only endogenous virus target included in this study, PMMoV showed relatively high persistence in wastewater solids compared to the other human respiratory viruses. The extended persistence of PMMoV has been reported previously both in wastewater solids (24) and other water matrices (47, 48), which suggests inherent characteristics of PMMoV might contribute to its high persistence. Future studies will need to more fully explore differences in the persistence of indigenous versus spiked viral RNA in wastewater solids for diverse human respiratory viruses.

Since there are no studies that we could find describing the decay of RNA from these respiratory viruses in wastewater solids, aside from the study by Roldan-Hernandez et al. (24) on SARS-CoV-2, it is not possible to compare our results to those of others. However, there are data on the decay of SARS-CoV-2 and IAV nucleic-acids in water and liquid wastewater matrices. For example, Atoui et al. (21) reviewed first-order decay rate constants of SARS-CoV-2 RNA in water and wastewater and found a wide *k* range from 0.040 to 4.3 day^−1^ depending on the experimental temperatures; the *k* values we report for wastewater solids range from 0 to 0.13 day^−1^ depending on temperature. Dovas et al. (49) reported avian influenza virus H5N1 RNA *k* values of 0.039–0.50 day^−1^ in various water matrices including river water, lagoon water, brackish water, and double distilled water at 4°C; our *k* values for IAV RNA in wastewater solids at 4°C are 0. From these limited comparisons, it seems that *k* values of viral RNA in wastewater solids are smaller than those observed in these studies which might suggest that the solids protect nucleic-acids through partitioning (50, 51). However, further research is needed to thoroughly investigate and understand this protective mechanism.

The data reported herein indicates the remarkable stability of short segments of viral genomic RNA from respiratory viruses in wastewater solids. The viral RNA in wastewater solids may be encapsulated within a virion, reside in a partially degraded virus capsid, or exist as naked RNA without the protection of a capsid and a lipid membrane (if enveloped) (52, 53). A study on wastewater containing SARS-CoV-2 suggests that a predominant portion of the detected SARS-CoV-2 nucleic-acid exists in intact, enveloped, but non-infectious particles (52). Harrison et al. (50) found that genomic nucleic-acids of bacteriophage MS2 and T4 were much more persistent when they were encapsulated in a virion. While we did not specifically examine the encapsulation status of the viral RNA in our study throughout the experiment, we suspect that it may have persisted encapsulated, considering the prolonged RNA persistence observed herein. Although beyond the scope of the present study, we recommend further research to investigate the relative occurrence of encapsulated versus non-encapsulated viral nucleic-acids in wastewater and wastewater solids, and their differential persistence. Such work could include the use of propidium-monoazide (RT-)PCR methods (53), the use of detergents to disrupt lipid envelopes (52), or nuclease to degrade unprotected viral nucleic-acids (54). Additionally, since the RT-ddPCR used herein only quantifies a short segment of the viral nucleic-acids, a more comprehensive analysis of viral nucleic-acid damage (55) may provide insights into the mechanisms of genome decay in wastewater solids.

A limitation of this study is that it was conducted using a single wastewater solids sample. The decay of viral nucleic-acids may be influenced by various physical, chemical, and biological attributes of wastewater, such as temperature, pH, solids content, detergents, organic materials, and microbial activity (41, 42, 55). A previous study by Roldan-Hernandez et al. (24) observed slight differences in the first-order decay of SARS-CoV-2 RNA in wastewater solids from two distinct treatment plants, potentially attributable to variations in the characteristics of the wastewater matrix. Consequently, future investigations should delve deeper into understanding the impact of wastewater solids’ characteristics on the decay of viral genomic materials, necessitating the consideration of seasonal and spatial variations in wastewater solids samples.

Another important limitation of the study is that we followed the decay of heat- and chemical-inactivated, exogenous viruses spiked into the samples. We chose to spike the solids sample with exogenous viruses because the endogenous virus nucleic-acid concentrations were low, and we wanted to be sure that we could document decay, should it occur. Future work on the decay of endogenous viruses would be particularly helpful. Such work would need to collect solids samples during periods when there is a high level of disease occurrence (and therefore viral shedding) in the community served by the wastewater plant. The use of inactivated viruses was a biosafety necessity for this work. It is difficult to know how the inactivation of the viruses affected the decay of their nucleic-acids in wastewater solids, particularly since there is still uncertainty regarding the state of the virus nucleic-acids in wastewater solids (present in intact or not-intact, infectious, or not infectious viruses).

## SUPPLEMENTAL MATERIAL

Supplemental material aem.02272-23-s0001.pdf Tables S1 to S7; Fig. S1.

ASM does not own the copyrights to Supplemental Material that may be linked to, or accessed through, an article. The authors have granted ASM a non-exclusive, world-wide license to publish the Supplemental Material files. Please contact the corresponding author directly for reuse.
