# Supplementary material for: Persistence of human respiratory viral RNA in wastewater-settled solids
Source: Appl Environ Microbiol. 2024 Mar 19;90(4):e02272-23. doi: 10.1128/aem.02272-23 (PMC11022535; doi:10.1128/aem.02272-23)
Supplement: Supplemental material — Tables S1 to S7; Fig. S1. [file aem.02272-23-s0001.pdf]

Persistence of Human Respiratory Viral RNA in Wastewater Settled Solids

Supplemental Material

Mengyang Zhang,[a] Laura Roldan-Hernandez,[a] Alexandria Boehm[a]#

[a] Department of Civil and Environmental Engineering, School of Engineering and Doerr School of Sustainability, Stanford University, Stanford, CA 94305, United States

#Address correspondence to Alexandria Boehm, aboehm@stanford.edu

Table S1. Percent of solids in wastewater solids sample collected from San José–Santa Clara Regional Wastewater Facility.

Data	Wet solids (g)	Dry solids (g)	Percent of solids
06/03/2023	0.3279	0.0502	0.1531

Table S2. Sequence information for primer and probe. bp represents base pair. FAM: fluorescent dye Fluorescein Amidite; HEX: fluorescent dye Hexachloro-fluorescein; IBFQ: Iowa Black FQ Quencher. ATCC (Manassas, Virginia); TWIST (South San Francisco, California); ZeptoMetrix (Buffalo, New York).

Virus	Target Gene	Sequence	Positive Control	Amplicon Size (bp)	Reference
SARS-CoV-2	N gene	Forward primer: 5′-CATTACGTTTGGTGGACCCT-3' Reverse primer: 5′-CCTTGCCATGTTGAGTGAGA-3' Probe: CGCGATCAAAACAACGTCGG (5’ FAM/ZEN/3’ IBFQ)	SARS-CoV-2 genomic RNA (ATCC VR-1986D)	143	1
HCoV - 229E	N gene	Forward primer: 5′-GGATGACATCATGAAGGCAG-3' Reverse primer: 5′-TACCCGTTTTCGCTGACTTT-3' Probe: TTCCTGAGGCTTGTCAAAACCT (5’ FAM/HEX/ZEN/3’ IBFQ)	Synthetic HCoV-229E gRNA (TWIST 103011)	92	2
HCoV - NL63	N gene	Forward primer: 5′-GAAGCGTGTTCCTACCAGAG-3' Reverse primer: 5′-TGGCATCAACACCATTCTGA-3' Probe: CAGTGCTTTGGTCCTCGTGA (5' HEX/ZEN/3' IBFQ)	Synthetic HCoV-NL63 gRNA (TWIST 103012)	107	2
HCoV - OC43	N gene	Forward primer: 5′-GTCTTTTACTCCTGGTAAGCAATC-3' Reverse primer: 5′-GGGTACAACATTCCCTCCTG-3' Probe: CCGATCAGTCCGACCAGTTTAG (5’ FAM/ZEN/3’ IBFQ)	Synthetic HCoV-OC43 RNA (TWIST 103013)	181	2
HRV	Untrans cribed region	Forward primer: 5′-GCCYGCGTGGCKGCC-3' Reverse primer: 5′-GAAACACGGACACCCAAAG-3' Probe: TCCTCCGGCCCCTGAATG (5’ HEX/ZEN/3’ IBFQ)	Intact virus (0810284CF, ZeptoMetrix)	204	2
RSV	N gene	Forward primer: 5′- CTCCAGAATAYAGGCATGAYTCTCC-3' Reverse primer: 5′-GCYCTYCTAATYACWGCTGTAAGAC-3' Probe: TAACCAAATTAGCAGCAGGAGATAGATCAG (5’ HEX/ZEN/3’ IBFQ)	Intact virus (0810040ACF, ZeptoMetrix)	121	3

Virus	Target Gene	Sequence	Positive Control	Amplicon Size (bp)	Reference
IAV	7 segment	Forward primer: 5′- CAAGACCAATCYTGTCACCTCTGAC -3'<br>Reverse primer: 5′- GCATTYTGGACAAAVCGTCTACG -3'<br>Probe: TGCAGTCCTCGCTCACTGGGCACG (5' FAM/ZEN/3' IBFQ)	IDT gene Block	106	[2]
PMMoV	ORF1	Forward primer: 5′-GAGTGGTTTGACCTTAACGTTTGA-3'<br>Reverse primer: 5′-TTGTCGGTTGCAATGCAAGT-3'<br>Probe: CCTACCGAAGCAAATG (5' FAM/ZEN/3' IBFQ)	IDT gene Block	68	[4]
BCoV	M gene	Forward primer: 5’-CTGGAAGTTGGTGGAGTT-3’<br>Reverse primer: 5’-ATTATCGGCCTAACATACATC-3’<br>Probe: CCTTCATATCTATACACATCAAGTTGTT (5' FAM/ZEN/3' IBFQ)	IDT gene Block	85	[5]

Table S3. Thermal cycling conditions for one-step RT-PCR for the detection of RNA targets from SARS-CoV-2, RSV, HCoV-OC43, HCoV-229E, HCoV-NL63, HRV, IAV, PMMoV, and BCoV.

<table>
<tr><th>Cycling Step</th><th>Temperature (°C)</th><th>Time</th><th>Number of Cycles</th></tr>
<tr><td>Reverse transcription</td><td>50</td><td>60 min</td><td>1</td></tr>
<tr><td>Enzyme activation</td><td>95</td><td>10 min</td><td>1</td></tr>
<tr><td>Denaturation</td><td>95</td><td>30 sec</td><td rowspan="2">40</td></tr>
<tr><td>Annealing/extension</td><td>HCoV-OC43, HCoV-229E, and HCoV-NL63: 59
IAV and HRV: 59
SARS-CoV-2 and RSV: 61
PMMoV: 56
BCoV: 56</td><td>1 min</td></tr>
<tr><td>Enzyme deactivation</td><td>98</td><td>10 min</td><td></td></tr>
<tr><td>Hold</td><td>4</td><td>Infinite</td><td>1</td></tr>
</table>

Table S4. BCoV recovery for each batch of RNA extraction.

Batch Number	Recovery (%)
1	114.74
2	81.48
3	55.86
4	56.29
5	113.41
6	91.90
7	83.24
8	108.10

Table S5. Coefficients for a multiple linear regression (equation 4 in the main manuscript), where effect of time, virus species and temperature on the natural logarithm decay of viral RNA was tested. The coefficients for nonsignificant terms ($p>0.05$) are not shown. The residual standard error for the model was 1.218 on 177 degrees of freedom, the adjusted R^2 was 0.5406, and $p<2.2\times10^{-16}$.

	Coefficient	Standard error	T statistic	p value
(Intercept)	1.14	0.31	3.672	0.000319
time	-0.037	0.01	-6.667	3.2×10^{-10}
PMMoV	1.95	0.36	5.408	2.1×10^{-7}
temperature	-0.077	0.01	-11.490	$<2.0\times10^{-16}$

Table S6. Post hoc Tukey contrasts among virus species pairs. Nonsignificant different pairs are not shown. Comparison indicates the hypothesis tested: that the difference in the decay ($\ln(C/C_0)$) between the named virus species is equal to zero. The Estimate is the estimated value of the difference. A positive Estimate value indicates the first virus listed decays more slowly than the second listed virus since the dependent variable is the natural log decay ($\ln(C/C_0)$) of viral RNA. $p<0.05$ represents a significant difference between the decay ($\ln(C/C_0)$) of named virus species.

Comparison	Estimate	Standard error	T statistic	p value
PMMOV - HCoV-229E == 0	1.94695	0.36005	5.408	<0.01
IAV - HCoV-NL63 == 0	1.15339	0.35546	3.245	0.030
PMMOV - HCoV-NL63 == 0	2.52939	0.35564	7.112	<0.01
PMMOV - HCoV-OC43 == 0	2.17065	0.36005	6.029	<0.01
PMMOV - HRV == 0	1.62758	0.35171	4.628	<0.01
PMMOV - IAV == 0	1.37600	0.35171	3.912	<0.01
RSV - PMMOV == 0	-1.98826	0.35171	-5.653	<0.01
SARS-CoV-2 - PMMOV == 0	-2.03791	0.35171	-5.794	<0.01

Table S7. Results from Tukey's honestly significant difference (HSD) test used for comparing the decay of two viruses at the same temperature (22 °C or 37 °C). Non significantly different pairs are not shown. "diff" represents the difference in the decay ($\ln(C/C_0)$) between the named two virus species in the column of "Comparison". A positive diff value indicates the first virus listed decays more slowly than the second listed virus since the dependent variable is the natural log decay ($\ln(C/C_0)$) of viral RNA. The "lwr" and "upr" provide the lower and upper bounds of the confidence interval at level of 95% for the difference. $p<0.05$ represents a significant difference between the decay ($\ln(C/C_0)$) of named virus species.

Temperature (°C)	Comparison	diff	lwr	upr	p value
22	PMMoV-HCoV-NL63	2.132336	1.322451	2.942222	0.0000000
	PMMoV-HCoV-OC43	1.936489	1.126604	2.746375	0.0000000
	PMMoV-HCoV-229E	1.711416	0.90153	2.521301	0.0000005
	RSV-PMMoV	-1.54756	-2.35744	-0.73767	0.0000053
	SARS-CoV-2-PMMoV	-1.47228	-2.28217	-0.6624	0.0000148
	HRV-HCoV-NL63	1.330993	0.521108	2.140879	0.0000994
	IAV-HCoV-NL63	1.167963	0.358077	1.977848	0.0008297
	HRV-HCoV-OC43	1.135146	0.325261	1.945032	0.0012541
	IAV-HCoV-OC43	0.972116	0.16223	1.782001	0.0088621
	PMMoV-IAV	0.964374	0.154488	1.774259	0.0096784
	HRV-HCoV-229E	0.910073	0.100187	1.719958	0.0176988
37	PMMoV-HCoV-NL63	4.901284	3.088446	6.714122	0.0000000
	PMMoV-HCoV-OC43	4.537884	2.641972	6.433795	0.0000000
	SARS-CoV-2-PMMoV	-4.17081	-5.91875	-2.42287	0.0000000
	RSV-PMMoV	-3.92149	-5.66944	-2.17355	0.0000002
	PMMoV-HRV	3.876165	2.128221	5.624109	0.0000002
	PMMoV-HCoV-229E	3.973717	2.077805	5.869628	0.0000009
	PMMoV-IAV	2.653921	0.905977	4.401865	0.0004095
	IAV-HCoV-NL63	2.247363	0.385615	4.109111	0.0085109

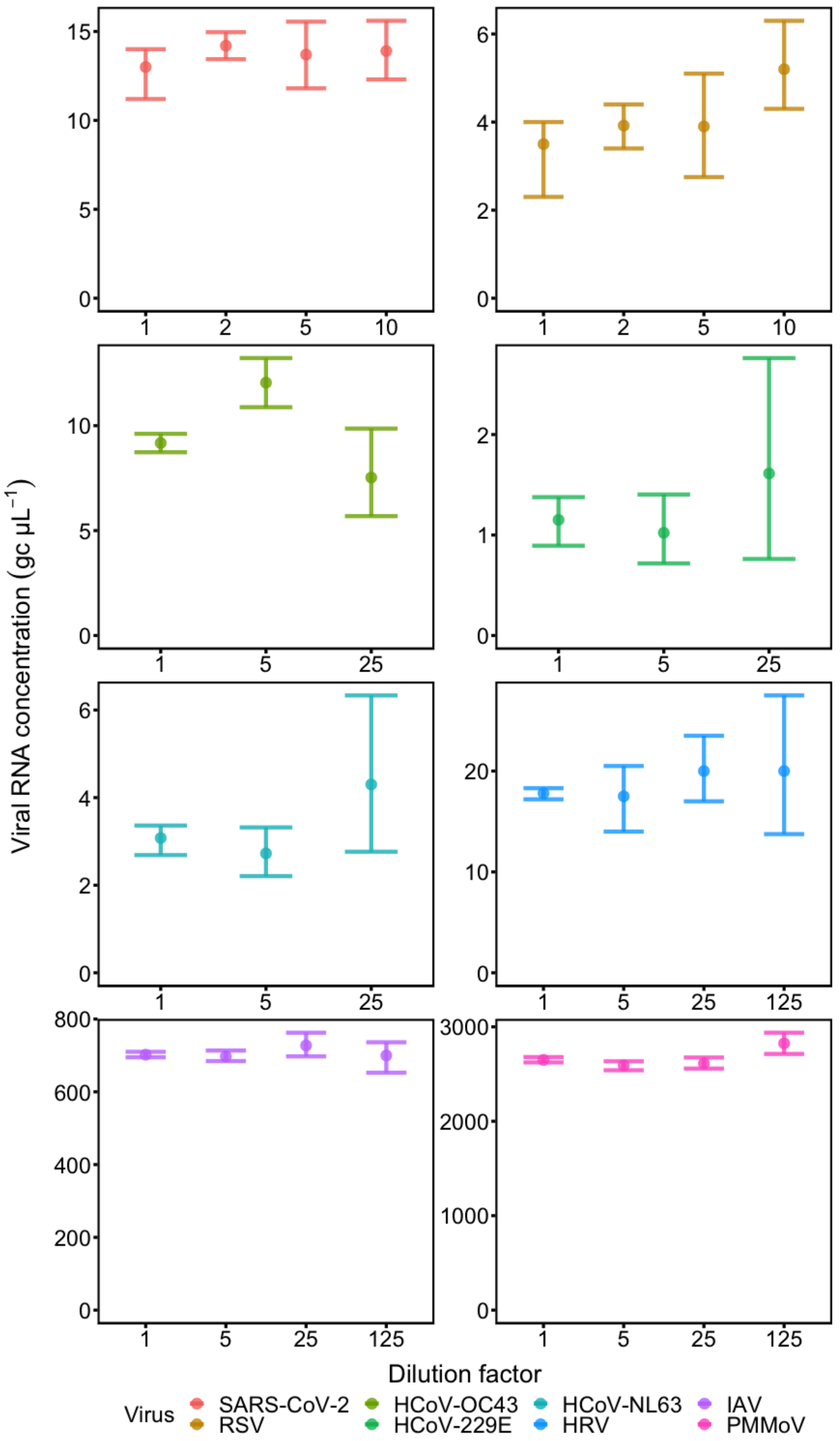

Figure S1. Inhibition assessment by a serial dilution of viral RNA template in RT-ddPCR. Viral RNA concentration refers to the concentration from QX Manager software, adjusted for dilution, with the unit of copies μL^{-1}, and the error bars represent the measurement standard deviation.

Reference

(1) Wolfe, M.; Hughes, B.; Duong, D.; Chan-Herur, V.; Wigginton, K. R.; White, B. J.; Boehm, A. B. Detection of SARS-CoV-2 Variants Mu, Beta, Gamma, Lambda, Delta, Alpha, and Omicron in Wastewater Settled Solids Using Mutation-Specific Assays Is Associated with Regional Detection of Variants in Clinical Samples. *Appl Environ Microbiol 88* (8), e00045-22. https://doi.org/10.1128/aem.00045-22.

(2) Boehm, A. B.; Hughes, B.; Duong, D.; Chan-Herur, V.; Buchman, A.; Wolfe, M. K.; White, B. J. Wastewater Concentrations of Human Influenza, Metapneumovirus, Parainfluenza, Respiratory Syncytial Virus, Rhinovirus, and Seasonal Coronavirus Nucleic-Acids during the COVID-19 Pandemic: A Surveillance Study. *Lancet Microbe* **2023**, *4* (5), e340–e348. https://doi.org/10.1016/S2666-5247(22)00386-X.

(3) Roldan-Hernandez, L.; Boehm, A. B. Adsorption of Respiratory Syncytial Virus, Rhinovirus, SARS-CoV-2, and F+ Bacteriophage MS2 RNA onto Wastewater Solids from Raw Wastewater. *Environ. Sci. Technol.* **2023**, *57* (36), 13346–13355. https://doi.org/10.1021/acs.est.3c03376.

(4) Haramoto, E.; Kitajima, M.; Kishida, N.; Konno, Y.; Katayama, H.; Asami, M.; Akiba, M. Occurrence of Pepper Mild Mottle Virus in Drinking Water Sources in Japan. *Applied and Environmental Microbiology* **2013**, *79* (23), 7413–7418. https://doi.org/10.1128/AEM.02354-13.

(5) Decaro, N.; Elia, G.; Campolo, M.; Desario, C.; Mari, V.; Radogna, A.; Colaianni, M. L.; Cirone, F.; Tempesta, M.; Buonavoglia, C. Detection of Bovine Coronavirus Using a TaqMan-Based Real-Time RT-PCR Assay. *Journal of Virological Methods* **2008**, *151* (2), 167–171. https://doi.org/10.1016/j.jviromet.2008.05.016.
